# Framework for Behavioral Analysis of Mobile Networks

**DOI:** 10.3390/s21103347

**Published:** 2021-05-12

**Authors:** José Antonio Trujillo, Isabel de-la-Bandera, David Palacios, Raquel Barco

**Affiliations:** 1 Department of Communications Engineering, Universidad de Málaga, Campus de Teatinos, 29071 Málaga, Spain; ibanderac@ic.uma.es (I.d.-l.-B.); rbm@ic.uma.es (R.B.); 2 Tupl Spain, Tupl Inc., Campus de Teatinos, 29071 Málaga, Spain; david.palacios@tupl.com

**Keywords:** mobile communication networks, cell behavior, Self-Organizing Maps (SOM), Random Forest

## Abstract

The arrival of the Fifth Generation (5G) entails a significant evolution in the context of mobile communication networks. This new technology will bring heterogeneous scenarios with new types of services and an increasingly high number of users and nodes. The efficient management of such complex networks has become an important challenge. To address this problem, automatic and efficient algorithms must be developed to facilitate operators’ management and optimization of their networks. These algorithms must be able to cope with a very high number of heterogeneous data and different types of scenarios. In this paper, a novel framework for a cellular network behavioral analysis and monitoring is presented. This framework is based on a combination of unsupervised and supervised machine learning techniques. The proposed system can analyze the behavior of cells and monitor them, searching for behavior changes over time. The information extracted by the framework can be used to improve subsequent management and optimization functions.

## 1. Introduction

The incoming Fifth Generation (5G) [1] networks are envisioned to overcome the boundaries of current Long-Term Evolution (LTE) networks, i.e., higher data rates, lower latency, reliable communications, increased number of connected devices or user coverage. In order to achieve this, new architecture and radio access network are standardized by 3rd Generation Partnership Project (3GPP) [2,3], enabling new services and scenarios in mobile communications [4]. Among these new services, the most noteworthy are: enhanced Mobile Broadband (eMBB) which is an improvement on current LTE data connections in terms of data rates and bandwidth; Ultra-Reliable Low Latency Communications (URLLC), which is intended for scenarios where reliability and latency are critical; and lastly, Massive Machine Type Communications (mMTC) which enables a scenario with a massive connection of devices, but with a lower volume of data exchange. The changes and improvements of this new 5G network mean an increase in the complexity of the network, and consequently in its management. In this context, Self-Organizing Networks (SON) [5] appear as a great alternative to automate the management of networks reducing the need of human intervention. SON functionalities include self-configuration, self-optimization, and self-healing methods to perform tasks related to network planning, performance improvement, and failure management, respectively. For the successful operation of these functions, certain key information gathered from different elements of the network, such as nodes or users, is required, such as network configuration parameters, network performance indicators, or context information. The amount of information available in networks is usually proportional to their complexity, so the high complexity expected in 5G networks with new scenarios and services will mean a large increase in the amount of data collected. However, a larger number of data does not always imply higher efficiency in SON tasks, as not all collected information turns out to be useful for the network management. For this reason, extracting as much useful information as possible from all available data is one of the challenges to be addressed to achieve an efficient performance of SONs.

To cope with this issue, Machine Learning (ML) algorithms [6] have become one of the most common alternatives. These techniques aim to learn from a set of data, extracting the useful information. A typical classification of ML methods divides them into supervised algorithms, which require labeled data (i.e., the dataset includes information about the state of the network at the time the data was collected) and unsupervised algorithms, which are able to learn from an unlabeled dataset [7]. These techniques have already been widely applied in mobile networks, so their application to 5G networks is expected to be essential for the automation of management tasks. Among these types of ML techniques, unsupervised methods present as an advantage that new network states may be found for dynamic scenarios that adapt to the new services of this new technology. Supervised methods usually achieve better results than unsupervised ones, but data in mobile networks are unlabeled in most cases.

In this paper, a framework for extracting useful information from a mobile network based on ML is proposed. Specifically, the system detects, classifies and monitors cell behavior patterns from an unlabeled dataset. For this purpose, the Self-Organizing Maps (SOM) [8] algorithm was firstly employed as an unsupervised algorithm, which clusters cells with similar behavior in the same group. Then, a supervised algorithm, namely Random Forest [9], is applied, to perform cell behavior classifications and monitoring in real time. As described in the next section, there are works in the literature that propose methods for cell classification or information extraction applied to mobile networks. However, most of them present important limitations. Some works are only focused on specific aspects of the network such as a certain type of traffic. Other works are limited to the analysis of historical data but do not include a real-time stage for network monitoring. Finally, many of them are based on only one ML method, not taking advantage of the benefits of combining unsupervised and supervised algorithms. In this context, this work provides the following contributions:

- The proposed framework is designed to analyze the overall performance in a mobile network, since it allows the inclusion of indicators related to different aspects of the network (e.g., traffic, radio conditions, quality). In addition, it can be set up for the characterization of the network focusing on a specific aspect such as type of traffic or a certain management task.
- The use of an unsupervised pre-stage allows the system to automatically tag an untagged real dataset for use as input to a subsequent supervised stage. This aspect enables the benefits of unsupervised and supervised algorithms to be exploited in the same system.
- The framework consists of two phases: a first training stage intended to acquire knowledge as cell patterns from the data and a second part that monitors the network to detect possible pattern change, not necessarily related to a network failure. For this reason, the proposed framework is intended to be used not only for information extraction, but also for network monitoring.
- The system has been tested with a live LTE dataset since it is currently the predominant technology. However, the methodology followed for the framework definition and implementation can be adapted to any other mobile network technology, since the SOM algorithm is based on finding patterns in the set of network indicators used as inputs regardless of the network technology. This amount of data is expected to be larger in the new 5G networks; hence, the use of the propose framework will benefit operators in the management tasks.

The rest of the paper is structured as follows. Section 2 reviews related work. Section 3 presents the proposed framework with definition and implementation details. Section 4 explains the experiments and results of a live LTE network. Finally, Section 5 describes the main conclusions of the work.

## 2. State of the Art

The concept of SON, standardized by 3GPP from the definition of LTE onwards [10], has been widely researched in the area of mobile networks. The last releases of the standard have included SON functionalities addressing the potential applications of SON tasks in the new 5G networks [11]. The emerging scenarios enabled by 5G networks are diverse, ranging from emergency situations and telemedicine [12] to massive connection of IoT devices [13]. In this context, the application of SON functionalities to 5G networks is a key challenge to automate optimization and management tasks [14]. In the literature, many works propose methods to implement these SON functions. Most of them are based on ML algorithms, although automatic methods such as fuzzy or rule-based systems are also proposed. In [15], ML alternatives for management tasks in mobile networks are analyzed in detail. In particular, the authors study alternatives for automating tasks such as anomaly detection, diagnosis and compensation, combining them in a system for fault management in mobile networks. The main contributions to the different SON tasks found in the literature are discussed below.

One of the main challenges in mobile networks is to achieve an efficient use of radio resources due to the limitation of availability. Hence, self-optimization is one of the most requested functionalities of SON, frequently mentioned in the literature. An approach for the optimization and management of radio resources in mobile networks is proposed in [16]. Based on network level, user level or user requirement, the best resource allocation in LTE and 5G networks is recommended. The authors of [17] develop an ML-based framework for optimizing network parameters to improve resource allocation and Quality of Experience (QoE). In [18], a system for optimizing handovers based on the SOM and K-Means is suggested. Parameters such as Time to Trigger (TTT) or handover hysteresis are modified to avoid unnecessary handovers between indoor and outdoor cells.

In the context of self-healing, anomaly detection is one of the most common tasks in mobile networks, enabling automatic detection of network failures. The main objective of anomaly detection methods in mobile networks is to identify anomalous states or faults in the cells. Some approaches are proposed in [19,20], which perform an SOM-based algorithm for the detection of faulty cells in mobile networks. The authors of [21] provide an anomaly detection system based on the Merge Growing Neural Gas (MGNG) algorithm. This algorithm uses data of Performance Indicators (PI) from the cells. Some works are focused on specific faults such as the system described in [22] for detecting sleeping cells and sudden increase in traffic. The authors implement a semi-supervised system that exploits statistical values to perform the detection. An automatic algorithm that aims to detect cell outages based on handover statistics is defined in [23].

Anomaly detection focuses on finding operational faults in the network, whereas diagnosis (also called Root Cause Analysis) tries to find the cause of the fault. Many works for automating the diagnosis task can also be found in the literature. A tool for diagnosis based on techniques such as tree classification and correlation is developed in [24]. In contrast to other systems, data are collected on a per-UE basis. It diagnoses the most common radio causes in an LTE network based on user throughput metrics. An automatic diagnosis system is implemented in [25] based on unsupervised ML techniques (SOM and Ward’s Hierarchical). In addition, expert knowledge is included to get more accurate results. The authors of [26] develop a framework to automate the diagnosis of radio conditions in the cells of a mobile network. The system is implemented with an SOM algorithm and uses user traces as inputs.

Lastly, prediction enables the anticipation of possible failures that may occur in the network. Thus, this task mitigates failures beforehand, although it is difficult to automate and achieve accurate results. The authors of [27] review the most common ML techniques and their possible application to automatic prediction. In [28], an ML-based approach to prediction is presented. The aim is to predict uplink transmission power levels using quality indicators and application level information. The authors of [29] propose a system for forecasting received signal levels. The approach is based on the supervised Random Forest algorithm.

As the complexity of the network increases, the number of data generated and collected increases significantly. A key factor in reaching suitable effectiveness in SON tasks consists in extracting useful information from a huge number of data. Characterization of the network through behavioral patterns is one of the most common methods of obtaining such useful information. In the area of mobile networks, several alternatives based on unsupervised ML algorithms are proposed, due to the fact that data collected in these networks are usually unlabeled. The authors of [30] base their proposal on the SOM algorithm to describe the global behavior of 3G networks. The SOM algorithm is also the basis of the framework proposed in [31], which also includes K-Means to fit the patterns detected by SOM. This combination is also implemented by the authors of [32] to classify behavior patterns of the radio access network of a mobile network. Most of the studied works provide a global overview of the network behavior. However, there are other works that are focused on specific aspects of the network. An SOM-based approach for traffic characterization of a network is proposed in [33]. In [34], cells are classified according to network traffic using the Naive Bayes technique (which applies Bayes’ theorem). The proposed system is a radio technology independent of the network, covering GSM to the New Radio of 5G. A method for extracting different patterns of application traffic is described in [35], which is based on Random Forest. The authors of [36] also use Random Forest to detect traffic patterns at the application level, including the importance of attributes as inputs for the classification.

A brief summary of each state-of-the-art work is presented in Table 1, pointing out the algorithms used and the purpose. As described before and as Table 1 shows, most works that propose SON functions are based on automatic methods or ML techniques and even combine two or more of these techniques in the same system, such as [31,32]. In these two works, SOM and K-Means are included in the same approach to detect patterns of behavior. As Table 1 reflects, some works employs the same algorithms for different objectives. This is the case of SOM, which is used with different aims such as anomaly detection or cell pattern detection. Random Forest is also used with different purposes such as classification and prediction in different works. However, combining the advantages of supervised and unsupervised algorithms in the same system is not sufficiently explored in the literature, since there are only a few works that address this approach. The authors of [35] propose the use of supervised and unsupervised algorithms in the same system for a mobile network scenario for the analysis of a specific type of traffic data (i.e., traffic of apps). In this respect, our work provides the ability to combine the advantages of unsupervised and supervised algorithms for the analysis and classification of the performance of a whole network without focusing on a specific aspect (e.g., type of traffic, context information) of the network. In addition, although most of the last works included in Table 1 are focused on the extraction of useful information to improve SON tasks, they do not propose any system for the monitoring in real time of the cell’s behavior in order to detect possible changes.

## 3. Proposed Framework

**Figure 1:**
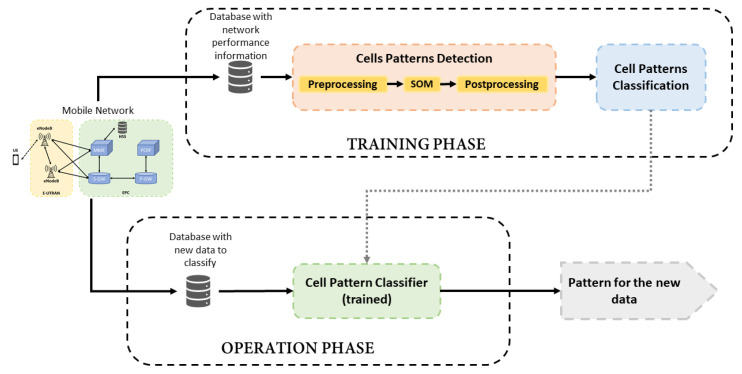
Block diagram of the proposed framework.

This section presents the details of the proposed framework for the classification and analysis of pattern behaviors in a mobile network. The system is composed of 2 parts: a first block that analyses and identifies the types of cell behavior from a network based on information collected during a given period of time, and another block that monitors the network gathering updated information to detect changes in cellsbehavior over time. Figure 1 shows an overview of the proposed system, where the two main parts of the framework are distinguished as Training and Operation phases. The training phase is divided into two functional blocks: Cell Patterns Detection and Cell Patterns Classification. The former is responsible for identifying the behavior patterns in the studied network from an unlabeled dataset and it is based on SOM algorithm [8]. As a result of the SOM application, the original dataset can be labeled to be used in the subsequent supervised stage. The second block, Cell Patterns Classification, is focused on the training of a Random Forest algorithm [9] with the labeled dataset in order to build a classifier. Thus, the training phase ends with the framework prepared for monitoring the cells.

Once information about cells behavior is extracted from the unlabeled dataset and the classifier is trained, the Operation Phase monitors the network to detect changes from new data collected.

The training phase needs a dataset large enough to detect reliable behavior patterns. It is executed at the beginning of the framework application and might be executed again after long periods of time to find new patterns of behavior in the network. On the other hand, the Operation Phase can be executed with certain periodicity, such as daily, weekly or monthly according to the operator’s preferences. Each element of the framework is described in detail in the following sections.

### 3.1. Training Phase

As described before, two functional blocks are defined as part of this phase: Cell Patterns Detection and Cells Patterns Classification.

**Figure 2:**
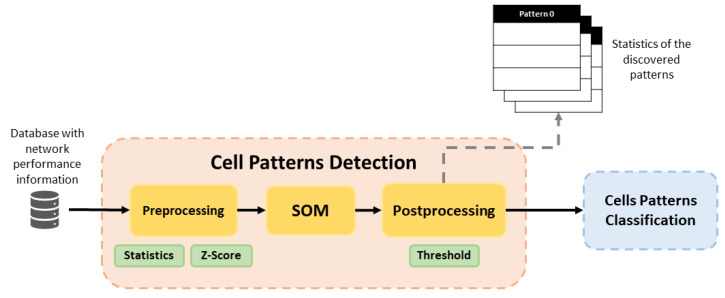
Block diagram of training phase in the proposed framework.

Figure 2 illustrates the training phase in detail, representing the flow followed by the data in this phase. Cell Pattern Detection block executes pre-processing for prepare data for the SOM. Then, the SOM detects behavior patterns and Post-Processing assigns a label to those cells with similar behavior pattern. In addition, a set of statistics and graphics are provided for each detected pattern in the dataset. In this way, a network operator can use this information to characterize features of each pattern, since the label assigned by post-processing is not descriptive, it simply associates similar cells. Cell Patterns Classification uses the trained Random Forest classifier to assign one of the identified cell patterns to each analyzed cell.

#### 3.1.1. Cell Patterns Detection Block

The SOM method is the main element of this block and it is executed as an unsupervised learning algorithm. Before the application of SOM, it is necessary to give the suitable format to the collected data in the pre-processing phase.

The system works with KPIs, which are usually collected in the form of time series. The pre-processing phase is responsible for characterizing the time series to some significant statistics. Both SOM and Random Forest will work with the same statistics, so the configuration of these is made at the beginning of the framework. Pre-processing also applies a normalization of the statistics in order to prevent some KPIs from having more importance than others in the classification. As the normalization method, *Z-Score* is used, which applies Equation (1) to each value to normalize,

(1) $$z=\frac{x-\mu }{\sigma }$$

where *z* is the normalized value, *x* the current value of a specific KPI, and μ and σ are the mean and standard deviation of the considered KPI, respectively. Once the data are normalized, a vector for each cell is generated, including the normalized statistics of each considered KPI. This vector will be the input of the SOM algorithm.

SOM is a widely used algorithm to cluster similar data from an unlabeled dataset, as discussed in the previous section. The ability of SOM to find similarities between different samples together with the absence of parameters that directly affect the number of clusters to be found by the algorithm, unlike K-Means or Canopy, are the main reasons for its preference over other unsupervised algorithms. Its operation is based on the construction of a two-dimensional map, where each point corresponds to a neuron. The concept of neuron in this algorithm represents the association between a point on the map and a weight vector, *W* = [*W*_1_,*W*_2_,*W*_3_⋯*W*_N_], which represents a cluster. These weight vectors can be initialized to certain values, although random initialization is often used. Then, in each iteration, the values of weight vectors are updated according to the input data and other configurable parameters. Several parameters decide the operation of the algorithm; therefore, these must be configured before starting the execution of SOM with initial values that can change during execution. Some of the most important parameters are:

- Learning rate (α). It indicates how much is learned from the input data in each iteration. It must be set to an initial value that will decrease with each iteration according to the decay function.
- Neighborhood rate (σ). It indicates which of the neurons on the map must be considered neighbors. It is interpreted as a maximum distance to the activated neuron in each iteration. Those neurons included in this distance are considered as neighbors and therefore update their weight vector. As the learning rate indicator, it must also be set to an initial value that will decrease with each iteration.
- Maximum of iterations. Indicates the maximum number of iterations that the algorithm performs if it does not reach convergence.
- Size of map. It is adjusted according to Expression (2),
  (2) $$x\cdot y≃5\cdot \sqrt{N}$$
  where *x* and *y* are the map dimensions and *N* is the number of input samples. The dimensions have to be chosen to get closer to the calculated value.
- Decay function. Function used to reduce learning and neighborhood rates in each iteration of the algorithm. The default function is shown in Expression (3),
  (3) $$x=\frac{x_{n}}{1+\frac{t}{T}}$$
  where $x_{n}$ is the new rate calculated and *x* is the rate of the previous iteration, *t* is the current iteration and *T* is half of maximal iterations allowed.

Once these parameters are configured, the steps that the algorithm will follow in each iteration during its execution are the following:

1. The Euclidean distances between each neuron and the input data are calculated. Weight vectors of each neuron are used together with the input weight vector following Expression (4),
  (4) $$E_{D}=\sqrt{\sum_{i=1}^{n}{\left(p_{i}-q_{i}\right)}^{2}}$$
  where *P = (p${}_{1}$,p${}_{2}$,p${}_{3}$,...,p${}_{n}$)* is the input vector and *Q = (q${}_{1}$,q${}_{2}$,q${}_{3}$,...,q${}_{n}$)* is each weight vector of the map. The BMU (*Best Matching Unit*) is the minimum distance between all those calculated, so the one with the shortest distance is considered the “winning neuron”.
2. The “winning neuron” must update its weight vector according to the learning rate and the input data vector.
3. Neurons considered as neighbors also update their weight vector. The neighborhood rate determines if a neuron is considered as a neighbor or not as indicated by Expression (5),
  (5) $$\left\{\begin{array}{ccc} 1\;\left(\mathrm{Neighbour}\right) & if & D<\sigma_{n}\\ 0\;(\mathrm{Not}\;\mathrm{Neighbour}) & if & D>\sigma_{n} \end{array}\right.$$
  where *D* is the distance of each neuron to the winner neuron and σ is the current neighborhood rate.
4. Before starting a new iteration, new values are calculated for the learning and neighborhood rates according to (6) and (7), respectively,
  (6) $$\alpha =\frac{\alpha (0)}{1+\frac{t}{T}}$$
  (7) $$\sigma =\frac{\sigma (0)}{1+\frac{t}{T}}$$
  where α(0) and σ(0) are the corresponding values of the previous iteration ( for this first iteration these values are the initial values) of learning rate and neighborhood rate, *t* is the current iteration and *T* is half of maximal iterations allowed. These new values are stored in α and σ so that new parameters can be used in steps 2 and 3 in the next iteration. Once updated, a new iteration can be started.

The execution of the algorithm concludes when the neighborhood and learning rates converge, i.e., if any of these parameters reaches 0, or if the configured maximum number of iterations is reached. The output of the algorithm will be a set of groups representing the different patterns of cells detected, and the cells belonging to each type.

Post-processing attempts to find similarities between the cell patterns detected by SOM. This stage is designed with the aim of reducing the initial amount of cell patterns detected by SOM, which may be high when a global vision of the network is required. Taking as reference the weight vectors trained by SOM, a calculation of the Euclidean distances between these weight vectors is performed. These distances are used to establish a threshold that limits which weights vectors are sufficiently similar to be considered the same behavior pattern. The threshold is calculated with Equation (8),

(8) $$Threshold=a\cdot \mu +b\cdot \sigma^{2}+c\cdot \sigma$$

where μ, $\sigma^{2}$ and y σ are mean, variance and standard deviation of the set of Euclidean distances calculated from the SOM weight vectors, respectively. On the other hand, a,b and *c* are the coefficients that give more or less importance to each statistic in the calculation of the threshold.

Once the threshold is computed, the Euclidean distances between each pair of weight vectors are calculated. The pair with the minimum distance is chosen, and it is compared to the threshold. If the distance is lower than the threshold, the neurons merge and a new weight vector is calculated using average values of previous vectors. The process is repeated until the minimum distance found between the neurons is higher than the threshold.

From here on, post-processing assigns a label to each behavior pattern, and then it tags each cell with its respective pattern. These associations are stored in a new labeled dataset that can be used by Random Forest in the Cell Patterns Classification block. Finally, the Cell Patterns Detection phase calculates some statistics for each studied KPI, and each cell pattern found after post-processing.

Finally, some graphics are plotted to show the identified behavior patterns and their differences. For both, data are organized by cells patterns and information is calculated per pattern. In this way, there will be an association among the behavior that defines each pattern that helps a network operator to know how each cell is working according to the pattern with which it has been labeled.

#### 3.1.2. Cell Pattern Classification Block

This block takes responsibility for building a classifier based on the Random Forest algorithm using the labeled dataset generated in Post-processing phase. The operation of Random Forest is explained below.

**Figure 3:**
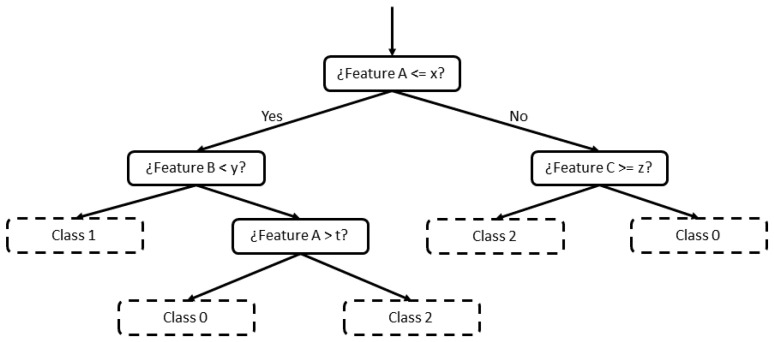
Decision tree example.

Random Forest [9] is a supervised ML algorithm based on decision trees. A decision tree is formed by a set of hierarchical nodes (also known as leaves), where each node is divided into two other nodes, in a downward direction. The criterion to decide each division is based on features of the input data (in this work, a feature would correspond to a KPI). Generally, the highest difference between two splits is searched comparing some features. An example of a decision tree is shown in Figure 3, where each node is divided into two until a value is reached for classification (nodes with dashed lines).

Random Forest is based on a pre-configured number of decision trees. Each of the trees make the classification independently, and Random Forest checks which is the most common classification among all the decision trees. Therefore, output of the algorithm is the classification most voted for. Furthermore, several parameters must be adjusted before starting the execution, related to the performance of both the decision trees and the complete set that builds the forest. Some of the most important configuration parameters are:

- Number of decision trees. It establishes the number of trees that constitutes the forest. It must be chosen in relation to the input dataset to avoid overhead.
- Division criterion. It defines how good a division is according to the condition set in the node. The two most commonly used criteria are: Gini and Entropy criteria. The Gini criterion measures the probability of failure if the classification is made in the current node, while Entropy measures the information gain that each division provides.
- Bootstrap. This parameter decides how each tree is built independently. If it is activated, the initial dataset is divided into different subsets, one for each tree. In this case, a maximum size for these subsets is established. Otherwise, the complete dataset is used for each tree.
- Maximum leaves per tree. It sets the limit of leaves on a tree in the forest.
- Minimum samples to split. It indicates the minimum of samples needed to consider a new split.
- Maximum samples to split. It indicates the maximum number of samples to consider in order to choose the condition that determines the split.

The operation of the algorithm has two parts, training and evaluation phases. Therefore, the input dataset is divided into two parts, a larger part for training and a smaller part for evaluation. In the training phase, the data are inserted into the algorithm with the corresponding label. In this way, the algorithm learns which values for each feature correspond to each label. In the evaluation phase, only the values of the different features are considered, and the algorithm provides a label as output. The output label is compared with the original label, thus calculating the accuracy of the trained classifier. Random forest is used for its ability to learn, as it achieves higher levels of accuracy with a lower number of data than other classifiers, as evidenced by the authors of [37].

### 3.2. Operation Phase

**Figure 4:**
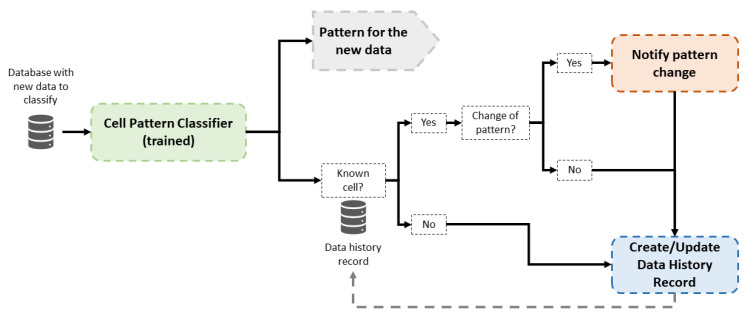
Block diagram of the operation phase in the proposed framework.

The operation phase has two main goals: the classification of new cell data and the detection of possible pattern changes in the behavior of previously classified cells. The operation of this part is presented in Figure 4. When new data are available, a classification is carried out by the trained Random Forest classifiers. Then, historical data are consulted to check whether the cell recently classified is known; that is, the cell has been classified previously in the system. In the affirmative case, the new classification is compared to historical data of this cell, and a notification is raised to inform a pattern change if classifications are different. In the negative case, data from this a new cell are stored with the assigned pattern classification. In this way, future pattern changes of this cell may be detected.

The behavior pattern of a certain cell can condition the operator’s decisions regarding tasks such as optimization or fault resolution. Therefore, information about the type of cell can help to implement more efficient management algorithms adapted to each type of network and cell. Moreover, network monitoring allows experts and operators to gain knowledge of network performance and use it to improve network management.

## 4. Experiments and Results

### 4.1. Experiments Setup

A battery of tests has been carried out to evaluate the performance of the proposed framework. Since it is difficult to find significant datasets from 5G networks, a pair of datasets of commercial LTE networks has been used. However, the proposed methodology is directly applicable to other technologies such as 5G. The largest LTE dataset is the one analyzed in more detail, and the results obtained with it are presented below. This dataset consists of 450 cells, where each one has 23 KPIs. These KPIs are related to different aspects of the cell such as throughput, traffic, quality or use of resources, among others. Table 2 shows the KPIs that have been included in the tests.

These KPIs are collected as a time series for a total of 46 days, with samples at hourly level. For the study carried out, weekends have been discarded, as well as holiday periods, resulting in a length of 25 days. From this time series, statistics are calculated to represent each KPI. In particular, the average, standard deviation, and 5th and 95th percentiles are calculated. If necessary, these statistics can be modified or completed it could be modified to include more metrics. Before SOM execution, statistics of each KPI are normalized by *Z-Score* normalization.

SOM algorithm is configured with the parameters of Table 3. The size of the map is adjusted to the 450 samples of the dataset, obtaining a total of 100 neurons in a 10 ×10 map. The initial neighborhood rate is set to 9, the maximum allowed by the size of the map. In addition, the learning rate is also set to a high value. A total of 100,000 iterations is established, which has been proven to be enough to get the algorithm to converge. The Batch training method is also established, which means that the samples are taken in the same order as in the dataset. The same decay function is maintained for both the learning and the neighborhood rate. Finally, other parameters of less relevance are kept at their default values.

Post-processing is configured by the coefficients assigned to each metric. Table 4 shows how high, medium and low thresholds are set.

After the execution of post-processing, a labeled dataset of 450 cells with 23 KPIs for each one and the type of each one is used. This dataset is divided for Cell Pattern Classification and Operation Phase. Specifically, 90% of the cells (405) are used to train Random Forest in the Cell Patterns Classification block and the remaining 10% (45) to test the effectiveness of the Operation Phase. In addition, the time series of the KPIs are divided into weeks for these parts of the framework instead of using them completely as in the Cell Patterns Detection. Therefore, the same statistics are calculated on a weekly basis.

In Cell Pattern Classification, the Cross-Validation [38] technique is used to get the best performance of the Random Forest. This technique makes different divisions of the dataset for the training and evaluation phases. In this way, the accuracy of Random Forest is more reliable given that it is calculated as average of accuracy of all splits. This accuracy is calculated as the coefficient of correct classifications and the total number of classifications. Once parameters of Random Forest are configured, training and evaluation phases are executed for each split of cross-validation. In the tests carried out, several values have been configured for cross-validation (5, 10, 15 and 20 splits).

### 4.2. Cell Pattern Detection

**Figure 5:**
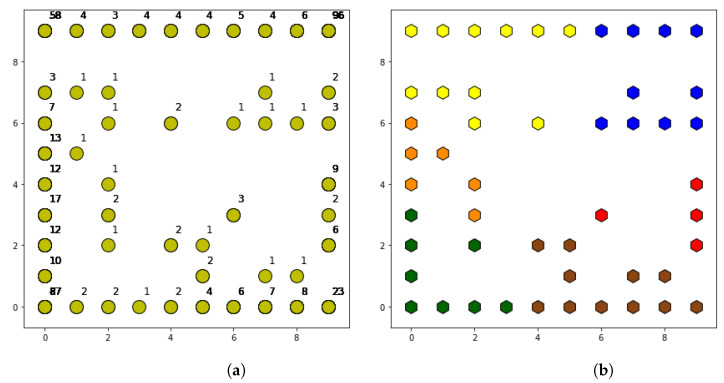
(**a**) Map of neurons at SOM output. (**b**) Map of neurons at post-processing output.

After SOM execution, a total of 50 different behavior patterns are identified as shown in Figure 5a. In the map shown in the figure, neurons that have been assigned at least one cell are distinguished by yellow dots. These are accompanied by a number that indicates the number of cells included in each group (i.e., the number of cells associated to the specific pattern). The map is built taking into account a neighborhood function, so that nearby neurons on the map have more similarities than neurons that are farther away. In this way, the corners of the map have the neurons with the highest number of cells, while intermediate patterns are found in the center of the map with the lowest number of accumulated cells. The limitation of this map lies in the large number of patterns detected, even if some of them only have a couple of cells. For this reason, it is necessary to have a post-processing phase that is capable of finding similarities between neighboring neurons, grouping together those that have fewer cells.

As shown in Table 5, when the post-processing is applied, a high threshold allows more neurons to join, since the limit distance is greater. As the threshold is reduced, the number of resulting patterns is greater, reaching 13 at the threshold, which is considered as low. The differences between thresholds are reflected in the level of detail of the obtained patterns. The four patterns obtained with a high threshold are more generic than the 13 obtained with the low threshold. Therefore, the configuration of the post-processing depends on the level of detail desired for the network behavior analysis.

Specifically, for a medium threshold, the obtained results are shown in Figure 5b. The shape of the map is the same as in the SOM output, but in this case the neurons have different colors. Each color represents one of the patterns obtained after post-processing, so the neurons with the same color are finally associated with the same cell pattern.

This set of patterns is analyzed in detail by comparing the KPIs of the cells associated with each behavior pattern. In Table 6, patterns found in the commercial network are characterized with the shown statistics.

- Pattern 0 (Green). This is one of the groups with more cells, and it could be considered as a good-behavior pattern for the analyzed network. It has a quite high number of connected users, and consequently a high number of transmitted data. Throughput per user and per cell achieves high values. It does not reach high levels of resource use, neither of data nor of signaling, and the blocking rate is quite low. It seems to represent not very large cells, which leads to lower latency. In addition, the connections quality is quite high.
- Pattern 1 (Red). This pattern represents a group of cells with a larger coverage area, although the number of connected users is not very high. The quality offered looks to be quite bad possibly due to high distance between users and base stations, which leads to a higher use of both signaling and data resources. However, the blocking rate is not too high. Poor quality also affects traffic levels and transmission rates on both the downlink and the uplink, despite the higher utilization of resources available in the cells.
- Pattern 2 (Blue). This pattern is the opposite of Pattern 0, and it characterizes those cells that may be performing worst in the network. These cells are of an intermediate size, but the number of connected users is very low. In addition, the high level of retries per connection indicates that users have trouble accessing the network. It also corresponds to the cells that offer the worst quality, and therefore the transmission levels achieved are quite low, as are the numbers of data transmitted on both links. The poor quality provided also appears in the use of resources, which presents values that are too high compared to the number of users in the cells. In addition, the blocking rate is quite high in this pattern despite not having a high number of users. In this case, there is not a clear reason to justify the behavior of this group, although it can be concluded that the performance of these cells is poor, and a configuration problem or external cause may be the reasons for this malfunction.
- Pattern 3 (Orange). This is an intermediate cell pattern because it reaches quite high-quality levels compared to those available for the cells corresponding to Pattern 0. The main difference is the number of users connected to the cells, which is slightly lower. This implies a lower use of both data and signaling resources. The size of the cells is also similar, even improving in terms of latency, as the transmission rates achieved per user are also better. It can be concluded that the cells belonging to this group have a good overall performance.
- Pattern 4 (Brown). This pattern is also in the middle, given that it is very similar to Pattern 0 in levels of connected users. In contrast, the quality offered by its cells is not as high as in Pattern 0; therefore, traffic levels and transmission rates on both links are affected. This quality, together with the larger size of the cells, leads to a higher use of available resources for both signaling and user data. In this case, as for Pattern 1, the high distance of connected users may be causing the decrease in quality in relation to other groups. In many cases, this high value of user distance is determined by a bad adjustment of some configuration parameters.
- Pattern 5 (Yellow). Finally, a pattern is identified whose quality is adequate, but the number of users connected is too low, with levels comparable to Pattern 2 being reached. In this way, the use of signaling and data resources is very low. Moreover, the volume of transmitted data is also very low in both directions. However, the transmission rates experienced by users are even better than in Pattern 0, as the quality is quite good, and the number of connected users is very low. These factors that cause the latency in the coverage area to be minimal, even if the coverage area is not particularly small.

### 4.3. Cell Patterns Classification

The patterns described in the previous section are used to train the Random Forest algorithm and build a pattern classifier. The results of accuracy for different values of Cross-Validation are shown in Table 7. It can be seen how the accuracy achieved by the classifier is very high with five splits. Despite the number of divisions being increased, the accuracy is similar given that it is already high enough.

This information is shown in more detail in Table 8, where results per each pattern are shown. In addition to the *accuracy* of each group, a couple of other statistics are calculated. *Recall* refers to the rate of positives with respect to the total, while *F1-Score* refers to the weighted average of *accuracy* and *Recall*. In most cases, the obtained results are quite high, since values are close to 1, except for Pattern 3, which has a *Recall* value below the average. The reason may be the similarity of this pattern with Pattern 5, which could lead to more misclassifications than expected.

In Table 9, the importance of each KPI in the classification of the cells is shown. For each KPI, the aggregate importance of the four statistics (Mean, standard deviation and 5th and 95th percentiles) used for the classification is calculated. Although there is a certain difference, it cannot be concluded that a certain KPI is greatly influencing the final classification given by the algorithm. Regarding the importance in the classification, some KPIs stand out from the rest; for instance, KPIs related to throughput both in uplink and downlink directions. However, there are others whose influence is very small due to the similarity of these in most groups. *Restablishments per connected user* is a good example because it is a KPI that is always 0, except in peaks, where the values reached are much higher. Another example is *Restablishment success rate*, since most of the time it is kept at values close to 100%, and it only suffers changes in certain moments.

Finally, the accumulative and average importances of the general categories are shown in Table 10. Some categories have a greater accumulative incidence (Traffic and Throughput, 43.79%) than others (Quality, 10.11% or Users, 11.55%). However, these categories include more KPIs than others, achieving higher values of percentage of importance. On the other hand, the average importance shows that no single KPI category is too influential in the classification of the behavior pattern. Thus, the highest average incidence is 7.29% (Traffic and Throughput), while the lowest is 2.37% (Other) with just 5% difference between them.

### 4.4. Operation Phase

**Figure 6:**
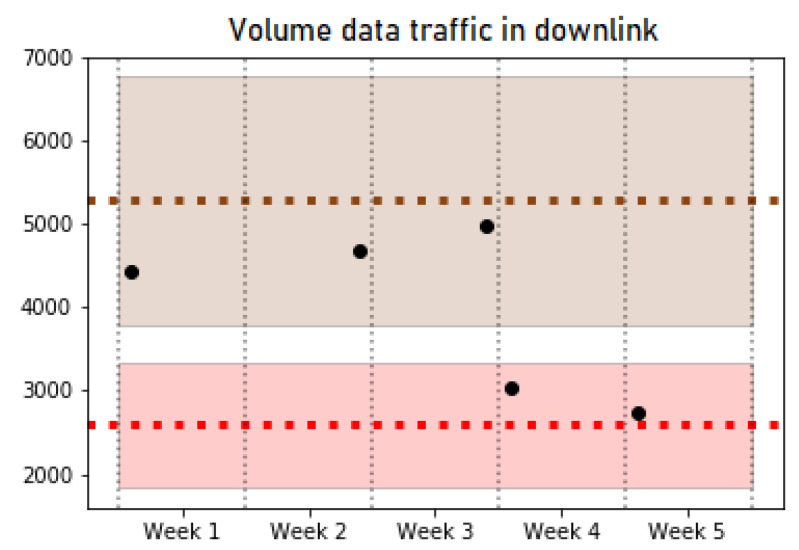
Volume data traffic in downlink.

**Figure 7:**
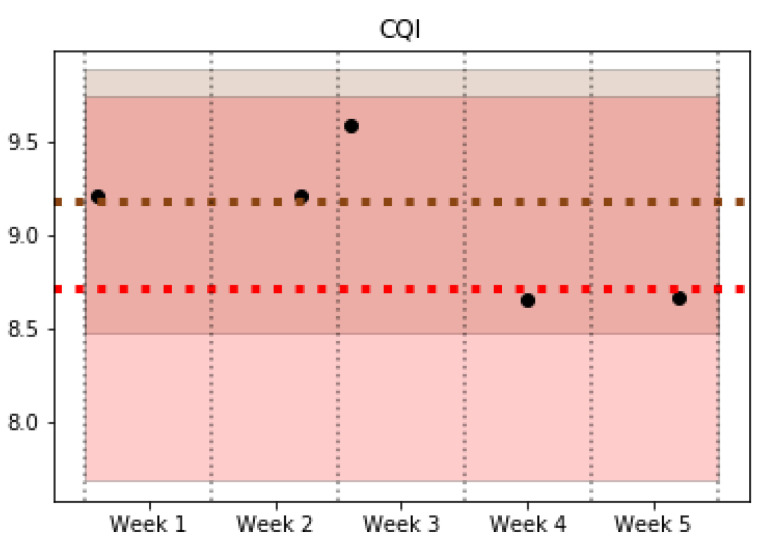
CQI.

In the operation phase, the trained classifier is used to find out the pattern that corresponds to new data collected from the network and to monitor possible changes in the pattern of a certain cell. Specifically, the tests carried out detected a total of nine pattern changes, of which 7 were sporadic cell behavior changes that return to their initial pattern after a short time period. However, the other two maintain the pattern change over time. Figure 6 and Figure 7 show examples of some of the KPIs for a specific cell that experience a behavior change from Pattern 1 (red) to Pattern 4 (brown). Both figures show a shaded area with the colors associated with Patterns 1 and 4, which highlights the typical values of the cells belonging to these patterns for a particular KPI. In addition, a couple of horizontal lines are included to indicate the average value of each pattern. The dots represent the average value of the cell studied in each week. The analyzed cell changes its pattern in the third week, and it maintains the new pattern in the last two weeks of data. On one hand, Figure 6 shows a KPI (*Data Traffic in Downlink*) where the change of pattern can be clearly observed. On the other hand, Figure 7 reflects the similar behavior in some KPIs such as *CQI*, which can be observed in two different patterns, making the decision to detect a pattern change. Nevertheless, the pattern change can be observed since the values of the cell per week take values closer to the average and deviation values (shaded) of Pattern 4 in the first 3 weeks (brown), while the last ones are closer to the values of Pattern 1 (red).

The tests were performed with the available dataset of a real LTE network, but the methodology followed for the implementation of the framework could be adapted with small changes to work with data from a 5G network. The pre-processing would undergo the main changes with the aim of adapting the data format of a 5G network to the format with which SOM has been implemented based on network performance indicator statistics. In the rest of the framework, no further significant changes would be required.

## 5. Conclusions

A framework for detecting and classifying behavior patterns in mobile networks has been presented. The system is independent of the mobile technology of the network under analysis, as it is based on a set of KPIs. First, an unsupervised learning stage, based on the SOM algorithm, enables the detection of different behavioral patterns of the analyzed network. In the second stage, a classifier based on a supervised learning algorithm (Random Forest) was constructed. Finally, the classifier was used to monitor the network and detect behavioral changes in the cells of the network.

The tests carried out show the good performance of the whole framework, analyzing the details of each stage of the system. The Cell Pattern Detection Block analyzed and extracted the behavioral patterns of a live LTE network, providing the inputs for the Cell Pattern Classification Block. The trained classifier shows results with good accuracy for the analyzed network dataset. These two blocks comprise the training part of the framework that can be configured to analyze the network with different levels of detail, i.e., to obtain more or less number of behavioral patterns of the network. Then, the framework enables the operator to monitor the network with a certain time period to detect behavioral changes. This was tested in the operation phase tests, where the cells were monitored every week and their behavior analyzed, detecting some changes between patterns.

In conclusion, the proposed framework provides an interesting tool to create a knowledge base of the analyzed network, thereby enabling the use of this knowledge to improve other management tasks in the network.

## Figures and Tables

**Table 1:** Summary of the main works in the literature.

Algorithms	Context/Objective	Work
Binary non-orthogonal Singular Value Decomposition (SVD)	Resource allocation optimization	[16]
XSOM (A modified SOM)	Handover management optimization	[18]
SOM	Anomaly detection	[19,20]
MGNG algorithm	Anomaly detection	[21]
Semi-supervised statistical-based algorithm	Sleeping cell detection	[22]
Rule-based system	Cell outage detection	[23]
Classification Tree	Diagnosis	[24]
Unsupervised techniques (SOM as the center-piece)	Diagnosis	[25]
SOM	Radio Frequencies (RF) conditions diagnosis	[26]
Random Forest, Deep Learning, Ridge Regression (Separated tests)	Transmission power prediction	[28]
Random Forest	Signal strength prediction	[29]
SOM	Cell pattern detection based on context information	[30]
SOM, K-Means	Cell pattern detection in 3G networks	[31]
SOM, K-Means	Radio access network analysis through behavioral patterns detection	[32]
SOM	Detection of daily traffic patterns	[33]
Naive Bayes, Holt-Winters	Classification of cells in terms of traffic	[34]
Unsupervised (Hierarchical clustering) and supervised (Random Forest) algorithms	Classification of traffic patterns by apps (Facebook, Twitter, Gmail, etc)	[35]
Improved Random Forest	Traffic pattern classification	[36]

**Table 2:** KPIs by categories.

Category	Key Performance Indicator
Traffic and Throughput	Cell throughput in downlink
	User throughput in downlink
	Volume data traffic in downlink
	Cell throughput in uplink
	User throughput in uplink
	Volume data traffic in uplink
Quality	QPSK usage rate in downlink
	QPSK usage rate in uplink
	Spectral efficiency downlink
	CQI
Users	Average of connected users
	Maximum of connected users
	Restablishments success rate
	Restablishments per connected user
Use of Resources	PRB usage rate in downlink
	PRB usage rate in uplink
	Blocking rate in signaling resources
	Signaling resources usage rate
Others	Latency in downlink
	RSSI in uplink control channel
	RSSI in uplink shared channel
	UE distance
	SINR lower than 2 dB rate

**Table 3:** SOM Configuration.

Parameter	Value
Size of map	10 × 10
Learning rate	0.9
neighbourhood rate	9
Iterations	100,000
Training method	*Batch*

**Table 4:** Thresholds in Post-processing.

Threshold	Coefficients
High	$0.5\cdot \mu +0.3\cdot \sigma^{2}+0.3\cdot \sigma$
Medium	$0.4\cdot \mu +0.25\cdot \sigma^{2}+0.25\cdot \sigma$
Low	$0.3\cdot \mu +0.2\cdot \sigma^{2}+0.2\cdot \sigma$

**Table 5:** Thresholds in Post-processing.

Threshold	Coefficients	Number of Patterns
High	$0.5\cdot \mu +0.3\cdot \sigma^{2}+0.3\cdot \sigma$	4
Medium	$0.4\cdot \mu +0.25\cdot \sigma^{2}+0.25\cdot \sigma$	6
Low	$0.3\cdot \mu +0.2\cdot \sigma^{2}+0.2\cdot \sigma$	13

**Table 6:** Patterns and statistics per KPI.

Key Performance Indicator	P0	P1	P2	P3	P4	P5
Number of cells	132	20	120	36	57	85
Cell throughput in downlink	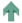	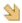	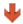	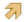	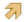	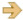
User throughput in downlink	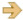	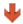	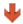	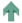	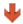	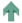
Volume data traffic in downlink	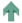	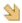	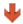	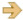	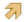	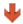
Cell throughput in uplink	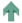	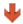	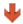	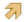	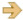	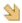
User throughput in uplink	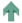	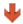	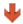	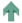	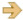	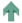
Volume data traffic in uplink	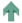	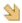	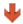	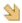	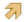	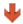
QPSK usage rate in downlink	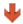	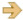	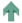	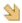	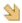	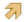
QPSK usage rate in uplink	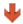	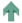	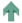	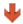	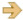	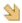
Spectral efficiency downlink	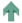	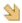	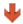	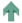	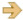	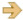
CQI	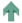	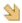	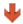	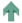	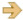	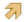
Average of connected users	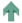	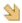	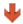	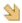	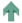	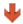
Maximum of connected users	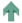	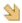	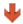	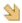	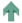	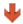
Restablishments success rate	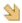	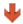	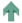	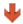	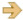	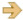
Restablishments per connected user	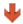	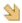	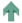	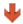	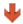	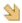
PRB usage rate in downlink	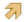	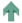	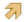	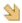	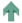	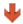
PRB usage rate in uplink	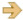	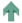	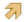	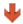	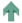	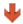
Blocking rate in signalling resources	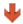	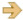	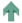	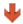	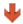	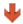
Signalling resources usage rate	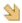	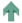	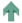	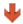	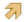	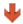
Latency in downlink	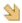	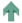	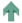	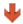	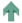	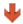
RSSI in uplink control channel	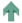	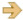	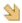	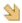	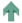	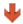
RSSI in uplink shared channel	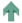	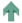	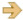	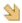	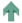	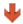
UE distance	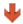	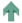	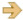	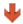	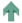	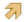
SINR lower than 2 dB rate	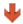	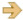	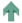	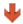	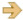	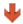

**Table 7:** Accuracy in the classification phase.

Cross-Validation	Accuracy
5	97% (±2%)
10	97% (±2%)
15	97% (±3%)
20	97% (±3%)

**Table 8:** Accuracy per pattern.

Pattern	Accuracy	Recall	F1-Score	Samples
**P0**	0.95	0.98	0.97	57
**P1**	1.00	0.89	0.94	9
**P2**	0.97	0.98	0.98	61
**P3**	1.00	0.79	0.88	19
**P4**	0.95	0.95	0.95	21
**P5**	0.92	0.97	0.97	36

**Table 9:** Importances of each KPI in classification.

Key Performance Indicator	Importance (%)
Cell throughput in downlink	11.00%
User throughput in downlink	2.92%
Volume data traffic in downlink	11.73%
Cell throughput in uplink	7.35%
User throughput in uplink	2.11%
Volume data traffic in uplink	8.67%
QPSK usage rate in downlink	2.34%
QPSK usage rate in uplink	3.51%
Spectral efficiency downlink	2.27%
CQI	2.00%
Average of connected users	5.14%
Maximum of connected users	4.18%
Restablishments success rate	1.32%
Restablishments per connected user	0.91%
PRB usage rate in downlink	3.16%
PRB usage rate in uplink	3.41%
Blocking rate in signalling resources	10.92%
Signalling resources usage rate	5.81%
Latency in downlink	4.29%
RSSI in uplink control channel	1.80%
RSSI in uplink shared channel	2.24%
UE distance	1.22%
SINR lower than 2 dB rate	1.69%

**Table 10:** Importances per category of KPI.

Category	Accumulated (%)	Average (%)
Traffic and Throughput	43.79	7.29
Quality	10.11	2.53
Users	11.55	2.88
Use of resources	23.3	5.82
Others	11.24	2.37

## Data Availability

Not applicable.
